# Contrast-induced acute kidney injury: a review of definition, pathogenesis, risk factors, prevention and treatment

**DOI:** 10.1186/s12882-024-03570-6

**Published:** 2024-04-22

**Authors:** Yanyan Li, Junda Wang

**Affiliations:** 1 Department of Pharmacy, Chongqing Traditional Chinese Medicine Hospital, 400021 Chongqing, P.R. China; 2 Department of Radiology, Chongqing Traditional Chinese Medicine Hospital, No. 6 Panxi 7 Branch Road, 400021 Chongqing, P.R. China

**Keywords:** Contrast-induced acute kidney injury, Pathogenesis, Review, Research progress

## Abstract

Contrast-induced acute kidney injury (CI-AKI) has become the third leading cause of hospital-acquired AKI, which seriously threatens the health of patients. To date, the precise pathogenesis of CI-AKI has remained not clear and may be related to the direct cytotoxicity, hypoxia and ischemia of medulla, and oxidative stress caused by iodine contrast medium, which have diverse physicochemical properties, including cytotoxicity, permeability and viscosity. The latest research shows that microRNAs (miRNAs) are also involved in apoptosis, pyroptosis, and autophagy which caused by iodine contrast medium (ICM), which may be implicated in the pathogenesis of CI-AKI. Unfortunately, effective therapy of CI-AKI is very limited at present. Therefore, effective prevention of CI-AKI is of great significance, and several preventive options, including hydration, antagonistic vasoconstriction, and antioxidant drugs, have been developed. Here, we review current knowledge about the features of iodine contrast medium, the definition, pathogenesis, molecular mechanism, risk factors, prevention and treatment of CI-AKI.

## Introduction

Contrast agents are injected into the body through blood vessels in order to change the image contrast of local tissues, enhance the development effect, as well as improve disease diagnosis and treatment. In recent years, with the extensive application of computed tomography CT in clinical diagnosis, iodine-containing contrast agents also known as iodine contrast medium (ICM) have become an indispensable part of disease diagnosis and have been widely used in clinical [1]. However, some studies have shown that 11–40% of patients with iodine contrast medium will develop acute kidney injury (AKI), that is, contrast-induced acute kidney injury (CI-AKI) or contrast induced nephropathy (CIN) [2, 3]. With in-depth research on the relationship between ICM and AKI, the term CIN has become less commonly used. CIN was a historic definition, replaced by CI-AKI, the term endorsed by Kidney Disease: Improving Global Outcomes (KDIGO) [4].

In order to distinguish and clarify the relationship between ICM and AKI, the European Society of Urogenital Radiology (ESUR) [5] and the American College of Radiology(ACR) [6]proposed the terms post-contrast kidney injury (PC-AKI), CI-AKI, and contrast-associated acute kidney injury (CA-AKI). The term PC-AKI is used to describe a decrease in renal function that follows intravascular administration of ICM within 48 h. CA-AKI: Any AKI occurring within 48 h after the administration of contrast media. The term PC-AKI is synonymous with CA-AKI and appears inradiology guidelines [6].Both terms imply correlative diagnosis. Neither term implies a causal relationship between contrast medium administration and an AKI event.CI-AKI is the subset of PC-AKI or CA-AKI that can be causally linked to contrast media administration. When it is clear that there is a causal relationship between ICM and AKI, it is recommended to use CI-AKI [5]. This review adopts the term CI-AKI.

CI-AKI has become a leading cause of hospital-acquired AKI affecting the health of patients in China. Hydration therapy was once considered the most convenient, effective, and economical method for preventing CI-AKI. Therefore, the 2012 KDIGO AKI Clinical Practice Guidelines [7] and the 2018 ESUR CI-AKI Prevention and Treatment Guidelines [8] both recommend the use of hydration therapy for the prevention of CI-AKI. Unfortunately,, there is currently no specific treatment for CI-AKI. Many randomized controlled trials (RCTs) and meta-analyses have analyzed various drugs such as N-acetylcysteine(NAC), statins, sodium glucose cotransporter 2 inhibitors (SGLT2i) and vitamin C, but there is no clear evidence to suggest that they can reduce the risk of CI-AKI [8, 9]. Effective treatment of CI-AKI is very limited at present. Therefore, the mechanisms underlying the occurrence and progression of CI-AKI have attracted much research attention in recent years. In this review, we highlight recent advances in our knowledge of CI-AKI pathogenesis, potential molecular mechanisms, risk factors, prevention and treatment of CI-AKI.

## Iodine contrast medium (ICM)

With the continuous advances of imaging technology, iodine contrast medium have been widely used in clinical diagnostic techniques, such as CT, commonly used for CT angiography and CT perfusion. And body cavity, joint, spinal cord imaging due to their abilities to improve the detection rate of lesion sites as well as facilitate lesion localization, qualitative and differential diagnosis [5]. The structure of iodine contrast medium used currently in clinical is a benzenic ring carrying three iodine ions, that is, one iodine ion is replaced in the 1, 3, 5 position of the benzenic ring, and the 2, 4, 6 position can bind three side chains, which together form a triiodobenzene ring derivative. According to the lonization state in solution, they can be divided into ionic and non-ionic contrast agents. As for non-ionic contrast agents, the non-ionic hydrophilic hydroxyl group is distributed around the benzene ring, shielding the hydrophobic iodophenyl group in it, thereby increasing the water solubility of the compound and reducing its toxicity [10]. Based on the size of plasma osmotic concentration, iodine contrast medium can be divided into high-osmolal, low-osmolal, and iso-osmolal ICM. The osmotic concentration of high-osmolal ICM is more than five times that of plasma osmotic; low-osmolal ICM is about two-three times that of plasma osmotic concentration (600–800 mmol/L); the osmotic concentration of iso-osmolal ICM is roughly the same as that of plasma osmotic concentration (about 290 mmol/L). According to the number of the benzene ring in the chemical structure, they can be divided into haplotype and dimer iodine contrast medium. Haplotype iodine contrast medium have only three iodine ions; the dimer iodine contrast medium are formed by connecting two triiodobenzene rings; thus, at the same iodine content, the dimer iodine contrast medium exhibit fewer molecule number, lower penetration concentration, and higher viscosity than the haplotype iodine contrast medium [11]. The details of iodine contrast medium are provided in Table 1.

**Table 1:** Classification and physicochemical properties of common iodine contrast medium

Classification	structure	Common name	Molecular weight	Iodine content(mg I/mL)	Osmotic pressure(mmol/L)
First generation (high-osmolal ICM)	Ionic haplotype	Meglumine diatrizoate	809	306	1530
Second generation (low-osmolal ICM)	Non-ionic haplotype	Iohexol	821	300	672
				350	844
		Iopamidol	777	300	616
				370	796
		Ioversol	807	300	651
				350	792
		Iopromide	791	300	607
				370	774
		Iomeprol	778	300	520
				350	620
Third generation (Iso-osmolal ICM)	Non-ionic dimer	Iodixanol	1550	270	290
				320	290

## CI-AKI definition

CI-AKI was first defined by the European Society of Urogenital Radiology (ESUR) in 2002: in the absence of surgery, nephrotoxic drugs and other factors, serum creatinine (Scr) content increased by 25% or 44.2 µmol/L compared with the baseline value within 72 h after intravascular administration of iodine contrast medium [12]. According to the updated 2011 ESUR Contrast Media Safety Committee guidelines, clinical CI-AKI is defined as the notion that without the influence of surgery, nephrotoxic drugs, and other causes, renal function is impaired, and Scr content is increased by 0.5 mg/dL (44.2 µmol/L) or more than 25% compared with the basic value within 72 h after intravascular injection of iodine contrast medium [13]. Although the KDIGO used the CI-AKI in 2012, it is defined as an absolute increase of 26.5 µmol/L in Scr content compared to baseline within 48 h after intravascular administration of iodine contrast medium, or a relative increase of more than 50% within 7 days, without the influence of factors such as surgery or nephrotoxic drugs [14]. The specialist consensus for CI-AKI in China indicates that exclusion of other factors, Scr levels increased by 25% or 0.5 mg/dL compared to the baseline value after use of contrast agents [15].

## Epidemiology

Approximately 13.3 million patients worldwide are diagnosed with AKI each year, of which 85% come from developing countries, and approximately 1.7 million deaths are associated with AKI each year [16].In addition to ischemic kidney injury caused by renal hypoperfusion or major surgery and drug-induced kidney injury, CI-AKI is the third leading cause of hospital-acquired AKI [17]. The risk of CI-AKI is significantly increased in elderly patients and patients with basic renal insufficiency, diabetes, and other adverse factors [18]. The incidence of CI-AKI in patients with chronic kidney disease is up to 40%. Because approximately 13% of hospitalized patients are likely to be permanently dependent on dialysis, CI-AKI is also associated with long-term kidney failure, hospitalization dialysis need, and overall mortality (7-31%) [19]. Among patients recovering from AKI, one-third of them will develop CKD within 2–5 years [20].

CI-AKI is associated with adverse clinical outcomes such as chronic renal failure and cardiovascular events. Although only 0.06% of patients require renal replacement therapy due to decreased renal function [21, 22], approximately 25–30% of CI-AKI will progress to chronic renal failure [23]; The average length of hospital stay and socio-economic burden of CI-AKI increase by 5–10 times [24].Therefore, CI-AKI is linked to longer hospital stay and higher cost, and has become an important disease affecting the health of patients.

## Pathogenesis of CI-AKI

**Fig. 1:**
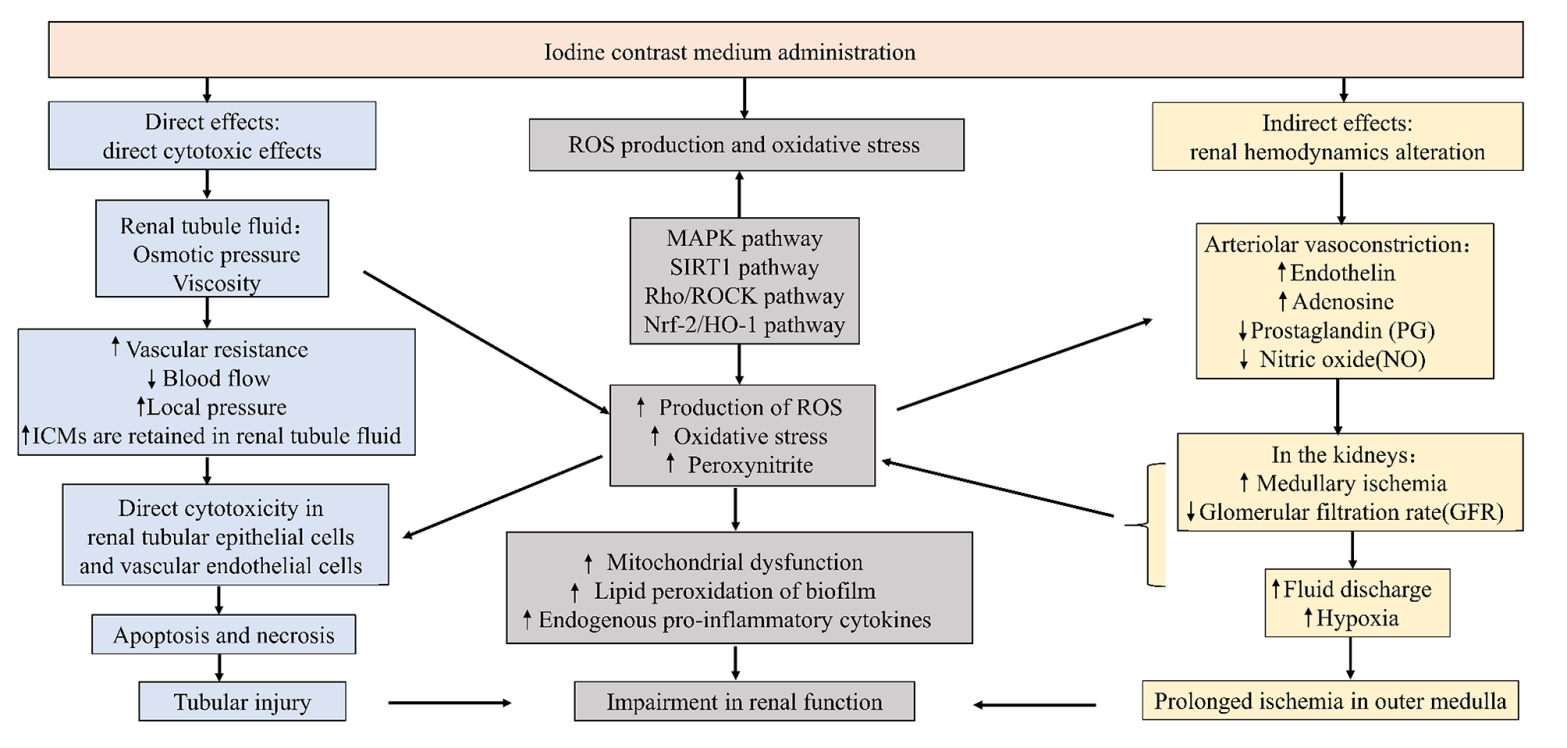
Pathogenesis of CI-AKI

The pathogenesis of CI-AKI is elusive, and several mechanisms, including contrast agents-induced direct effects, indirect effects and the production of reactive oxygen species (ROS), have been unveiled to be involved in the development of CI-AKI [25]. In the direct effects, iodine contrast medium can directly induce cytotoxicity of nephrons, including kidney tubular epithelial cells and endothelial cells, leading to mitochondrial dysfunction, apoptosis, pyroptosis, necrosis, and interstitial inflammation. In the indirect effects, iodine contrast medium can change the renal hemodynamics, thereby resulting in the contraction of renal blood vessels and intramedullary ischemia and hypoxia. On the other hand, iodine contrast medium can cause excessive production of ROS or reduce the activity of antioxidant enzymes to result in increased oxidative stress and inflammatory response, and thereby impairing renal function [26]. In addition, renal medullary hypoxia exacerbates ROS generation, which can lead to mitochondrial oxidative stress and mitochondrial dysfunction [27]. The pathogenesis of CI-AKI is provided in Fig. 1.

### Direct effects: direct cytotoxic effects of iodine contrast medium

Iodine contrast medium have direct cytotoxic effects on renal tubular epithelial cells and vascular endothelial cells, leading to swelling, vacuolation, apoptosis, and eventually necrosis, but the precise mechanisms are not fully understood. When these agents pass through the renal tubules, the water is re-absorbed and the contrast agents are concentrated, so the osmotic pressure and viscosity of renal tubule fluid are increased. The increase of viscosity is more obvious, and the vascular resistance is promoted by viscosity increase, leading to slowed blood flow and enhanced local pressure. Because of the increased viscosity, these agents are retained in renal tubule fluid and further result in direct cytotoxicity, thereby affecting mitochondrial enzyme activity and causing abnormal energy metabolism, cell calcium reduction, DNA breakage, mitochondrial dysfunction, and eventually cell apoptosis [28]. Mccullough et al. [29]pointed out that the highly permeable environment induced by the accumulated iodine contrast medium plays an important role in cell apoptosis. Zager et al. [30]revealed that the direct cytotoxic effects induced by these contrast agents largely depend on the extent of mitochondrial damage and plasma membrane damage. These necrotic tubular epithelial cells separate from the basement membrane, fall into the urinary lumen, and cause lumen obstruction and increase tubular pressure, which result in a decreased rate of glomerular filtration.

The cytotoxic effects of iodine contrast medium on renal tubular epithelial cells are characterized by vacuoles in renal tubular epithelial cells, excessive oxidative stress induced by increased ROS production, mitochondrial dysfunction, and subsequent ATP reduction. Vacuolation of renal tubular epithelial cells is a drug toxicity indicator of iodine contrast medium and a histopathological feature of CI-AKI [31]. Studies have shown that these contrast agents exert cytotoxic effects in vitro, and almost all types of cells, including vascular endothelial cells and renal tubular epithelial cells, present varying degrees of cell damage or apoptosis when exposure to these agents. Through electron microscope, these agents are found to cause vascular endothelial cell injury, shedding, vacuole formation, vascular endothelial barrier destruction, and permeability increase [32]. Huang et al. [33] reported that after iodine contrast medium injecteing into rat tail vein, these rats present swollen kidneys, marked area of congestion in the coronal cortical-medullary junction, increased tubular vacuolation and denaturation of renal tubular epithelial cells, many shed cells in the lumen, congested veins, and blurred cell boundaries, thus leading to CI-AKI development. The molecular mechanisms of cytotoxicity of these contrast agents may mainly involve Caspase-3, Caspase-9 and other pathways, which are directly linked to apoptosis signaling, overload intracellular calcium, reduced cell proliferation, and mitochondrial dysfunction [34, 35].

### Indirect effects: iodine contrast medium change renal hemodynamics

Renal medullary hypoxia caused by iodine contrast medium-imposed hemodynamic alterations is the leading cause of CI-AKI [36]. After intravascular administration of iodine contrast medium, several hemodynamic changes were observed in the kidneys: an initial rapid, transient hemangiectasis and an initial increase in renal blood flow (RBF); the subsequent sustained vasoconstriction, increased renal vascular resistance, and decreased RBF [37]. These hemodynamic alterations induced by iodine contrast medium may be attributed to the regulation of the synthesis and release of renal vasoactive factors [38]. To overcome the lack of oxygen supply induced by these contrast agents, many factors, such as prostaglandin (PG) and nitric oxide (NO), are generated and released to enhance blood flow. However, the direct cytotoxicity of iodine contrast medium to vascular endothelial cells results in an elevation in endothelin and adenosine levels and a decrease in NO and PG expression [39]. Once the balance between these two opposing effects is broken and shifts toward vasoconstriction, it can trigger medullary ischemia and a decline in glomerular filtration rate (GFR) in the kidneys, especially the outer medulla [40]. During ischemia, blood flow in the kidneys will shift from the medulla to the cortex, resulting in more severe medulla hypoxia [41]. Furthermore, iodine contrast medium also cause osmotic diuresis, which enhances fluid discharge and exacerbates hypoxia [42]. Notably, in patients with chronic kidney disease with reduced renal parenchyma, conventional doses of contrast agents can result in enhanced external medullary ischemia [36]. In addition, diabetic nephropathy is pathologically characterized by renal vascular dysfunction, which may trigger increased sensitivity of vasoconstriction induced by contrast agents, leading to an increased risk of CI-AKI [43, 44].

### ROS production and oxidative stress

Mitochondria are important sites for ROS production. During the process of ischemia and hypoxia, the balance of oxidative stress is disrupted, and mitochondria ROS (mtROS) is overproduced, which further causes lipid peroxidation of mitochondrial membrane and cell membrane, mitochondrial DNA damage, pyroptosis, and apoptosis [45]. ROS can also lead to direct cytotoxicity of renal tubular epithelial cells and vascular endothelial cells, and aggravate renal parenchymal hypoxia by inducing endothelial dysfunction, which together lead to a vicious cycle [27]. Damage-associated molecular patterns (DAMPs) and pathogen-associated molecular patterns(PAMPs) can produce ROS when stimulated [46]. In addition, DAMPs or PAMPs can combine with specific ligands innate immune cells express pattern recognition receptors (PRRs), such as toll-like receptors (TLRs) and NOD-like receptors (NLRs), which can activate some signaling pathways, such as MAPK, and promoting cytokine release. Some of these factors may be involved in CI-AKI [47, 48]. Work from the past decade has documented that iodine contrast medium induce ROS production mainly through the following four pathways [26].

#### Mitogen-activated protein kinase (MAPK) signaling pathway

This pathway consists of extracellular signal-associated kinase (ERK), c-JUN N-terminal kinase (JNK), and p38 MAPK. Iodine contrast medium activate the MAPK signaling pathway, as evidenced by activated ERK1, ERK2, JNK1, JNK2, JNK3, p38 MAPK, and ERK5 [49], to induce ROS production and then enhance the activity of Caspase-9 and Caspase-3, thereby contributing to apoptosis [45]. These contrast agents can also lead to cytotoxicity of human renal tubular epithelial HK-2 cells by repressing cell viability and causing severe mitochondrial vacuolar degeneration and karorrhexis [31]. In human embryonic kidney 293T cells, iodine contrast medium are capable of activating the JNK signaling pathway and thus induce ROS production and diminish cell viability. These studies suggest that the MAPK signaling pathway is involved in iodine contrast medium-induced ROS production [50].

#### Sirtuin 1 (SIRT1) signaling pathway

SIRT1 is a histone deacetylase of nicotinamide adenine dinucleotide (NAD+), which is mainly present in the nucleus [51]. In rat renal tubular epithelial NRK-52E cells, iodine contrast medium can result in increased ROS production and enhanced cell apoptosis by down-regulating SIRT1. Resveratrol attenuates contrast-induced nephrotoxicity in mice by diminishing oxidative stress and apoptosis by activating the SIRT1-PGC-1 Alpha FoxO1 pathway [52].

#### Rho/Rho-associated kinase (Rho/ROCK) signaling pathway

The Rho/ROCK pathway is an important regulator of vascular smooth muscle cell contraction, cell migration, proliferation, and differentiation. In mice, iodine contrast medium enhance the transcriptional activity of nuclear factor-κB (NF-κB), oxidative stress, inflammation, and apoptosis by activating the Rho/ROCK pathway and thus result in impaired renal function [53].

#### Nuclear factor erythroid 2-related factor 2/heme oxygenase-1 (Nrf-2/HO-1) signaling pathway

In a mouse model of CI-AKI, the Nrf-2/HO-1 pathway is involved in many cellular functions, including mitochondrial oxidative stress, autophagy, and programmed cell death [54]. Nrf-2 attenuates cell injury by inducing transcription of antioxidant enzyme genes, reducing ROS production, and diminishing oxidative stress. After administration of iodine contrast medium, the Nrf-2/HO-1 pathway is activated to produce an adaptive protective response that can mitigate contrast-induced tissue damage, oxidative stress, and apoptosis [55, 56]. In HK-2 cells, sulfamefen, an activator of the Nrf-2, can reduce ROS content and increase cell viability [57].

Collectively, iodine contrast medium can result in decreased blood flow and increased oxygen consumption in the kidney medulla by inducing hemodynamic alterations, and the resulting state of ischemia and hypoxia further enhances ROS production. Meantime, ROS production can be elevated by tubular necrosis or vacuolar degeneration of epithelial cells induced by the osmotic effect of iodine contrast medium [58]. In turn, the excessive production of ROS and the inactivation of NO synthase (NOS) and prostacyclin synthase cause contraction of renal vessels, and further aggravate the ischemia/hypoxia state in renal tissues. The excessive production of ROS also promotes oxidative stress and thus directly leads to dysfunction of renal tubular epithelial cells and endothelial cells [59], which together form a vicious cycle. Furthermore, the peroxynitrite with strong oxidative ability is generated by reaction of the superoxide anion of ROS and NO, which causes a greater damage to vascular endothelial cells [32]. Oxidative stress can also induce endothelial dysfunction and tubular transport dysfunction, resulting in dual effects of tubular oxidative injury and ischemic injury [45]. In addition, ROS-triggered oxidative stress can induce mitochondrial dysfunction, cell apoptosis, lipid peroxidation of biofilm, etc., and can promote the release of endogenous pro-inflammatory cytokines and activate the auto-inflammatory signaling pathways [60].

## Mechanism of the toxic effects of iodine contrast medium and the related ROS

The excessive production of ROS can abate iodine contrast medium-induced antioxidant enzyme activity and thus contributes to renal function impairment [61]. Studies have uncovered that several importantly cellular processes, including apoptosis, pyroptosis, as well as autophagy, and some epigenetic regulators, such as microRNAs (miRNAs), have implicated in the cytotoxicity effects of iodine contrast medium and the induced ROS [62]. The details of molecular mechanism are provided in Table 2.

### Apoptosis

Apoptosis, a kind of programmed cell death characterised by a series of irreversible intracellular changes, can be caused by the activation of Caspase family members induced by various pro-apoptotic stimuli, such as endoplasmic reticulum stress and ROS. The morphological manifestations of apoptosis include DNA fragmentation, cytoplasmic condensation, karyopycnosis, karyorrhexis, and apoptotic body formation. However, the cell membrane remains intact during the process of apoptosis, and thus no contents are released, which does not directly cause further inflammatory response [63].

Oxidative stress induced by ROS is a potent inducer of apoptosis. Apoptosis is involved in the pathogenesis of CI-AKI. Ca^2+^ overload in renal parenchyma is also found to play a crucial role in CI-AKI through ROS overproduction, p38 MAPK activation, as well as endothelin activation and release [64]. Studies have also shown that oxidative stress caused by ROS accumulation plays an important role in iohexol-induced AKI. Cilnidipine, a blocker of calcium channel, reduces ROS overproduction and alleviates iohexol-induced cell apoptosis, oxidative stress and mitochondrial damage of renal tubular cells in vivo and in vitro by blocking the CaMII-mPTP pathway and inhibiting Ca^2+^ overload [65].

### Pyroptosis

Pyroptosis, an inflammatory kind of cell death, is an important natural immune response characterized by cell swelling, cell membrane rupture, and the release of cell contents. Pro-inflammatory response is the most distinct and important characteristic that distinguishes it from apoptosis. Iodine contrast medium can strongly enhance host immune response and the release of inflammatory cytokines by inducing the destruction of tubular epithelial cell barrier as well as the injury and necrosis of endothelial cells. The NOD-like receptor pyrin protein 3 (NLRP3) inflammasome has been identified as a crucial regulator of inflammation. As a key component of innate immunity, the NLRP3 inflammasome plays an essential role in the immune response [66]. The NLRP3 inflammasome can be activated by various stimuli, including K^+^ efflux, mitochondrial ROS production, cathepsin release, as well as mitochondrial DNA and cardiolipin release [67]. In the kidney, this inflammasome can be activated by ROS [68]. Excessive ROS production induced by contrast agents is considered to be one of the key molecules in activating the NLRP3 inflammasome. In the resting state, the binding of thioredoxin (TXNIP) and thioredoxin (TRX) can maintain the dynamic balance of oxidation and antioxidant in the body. When ROS activation, TXNIP dissociates from TRX and then binds to the NLRP3 inflammasome, which further participates in oxidative stress and inflammation [69].

The classical NLRP3-Caspase-1-GSDMD inflammatory pathway plays a critical role in CI-AKI. Using in vivo and in vitro experiments, αKlotho, a protein with anti-inflammatory and antioxidant properties, alleviates contrast agents-induced pyroptosis by diminishing the release of GSDMD and IL-1β by significantly suppressing the activation of the NLRP3 inflammasome [70]. Chen et al. [71]uncovered that contrast agents cause kidney injury, up-regulate the protein expression of Caspase-1, NLRP3, ASC and GSDMD, and promote the release of IL-1β, IL-18 and lactate dehydrogenase (LDH), indicating that the classical NLRP3-Caspase-1-GSDMD pathway is implicated in CI-AKI pathogenesis.

In non-classical pyroptotic pathway, LPS enters the cytoplasm via transfection or infection and then activates Caspase-4, Caspase-5, and Caspase-11. Activated Caspase-4, Caspase-5, and Caspase-11 disrupt cell function and induce pyroptosis by forming pores in cell membrane by producing N-terminal p30 structure via GSDMD dissociation [72]. Zhang et al. [73] found that in contrast agents-induced AKI, inflammatory factors Caspase-4, Caspase-5, and Caspase-11 can mediate pyroptosis of renal tubular epithelial cells, leading to the occurrence of CI-AKI.

### Mitophagy

Autophagy supplies energy for cells, maintains self-repair ability and cell homeostasis, as well as removes defective protein molecules and organelles in cells. Autophagy is divided into three types: macroautophagy, microautophagy, and chaperon-mediated autophagy. Mitophagy is a unique process in which autophagosomes specifically swallow and degrade damaged mitochondria. Mitophagy is a mechanism for repairing damaged mitochondria, and its core function is to specifically clear damaged mitochondria and to control the number of mitochondria [74]. Mitochondria are the main source of ROS and the primary organelles for energy production and oxidative stress. Because of ischemia and toxic drug exposure, abnormal mitochondrial morphology and mitochondrial DNA mutations are found in AKI [75]. Damaged mitochondria will lead to the release of ROS and Cyt C, and subsequently induce apoptosis. Mitophagy is a method for the selective removal of damaged mitochondria, and it can protect cells from damage induced by excessive ROS [76]. These contrast agents can stimulate the renal tubular epithelial cells to produce excessive ROS and thus induce mitophagy [77]. Mitophagy is a selective autophagy mechanism that controls mitochondrial mass and mitochondrial ROS (mtROS) content through the degradation of damaged mitochondria. Studies have demonstrated that in CI-AKI, contrast agents can lead to mitochondrial damage and increased mitophagy by destroying the antioxidant defense mechanism, and mitophagy has a protective effect on the apoptosis of renal tubular epithelial cells [78]. Lin et al. [79] found that iohexol can cause mitochondrial damage in renal tubular epithelial cells in CI-AKI mice, thereby inducing the excessive production of mtROS and the activation of the NLRP3 inflammasome. The Pink1/Parkin pathway-mediated mitophagy can repair damaged mitochondria and attenuate the apoptosis of renal tubular epithelial cells and kidney injury in CI-AKI by reducing the production of mtROS and inhibiting the activation of the NLRP3 inflammasome.

Activation of autophagy can reduce the apoptosis of renal tubular epithelial cells induced by contrast agents, which may be related to the reduction of oxidative stress. Yang et al. [80]found that damaged mitochondria induced by contrast agents are the main source of ROS. The immunomodulator rapamycin reduces the apoptosis of renal tubular epithelial cells and protects against CI-AKI by up-regulating Parkin expression, enhancing mitophagy, alleviating mitochondrial damage and oxidative stress caused by ROS overproduction. In addition, melatonin can significantly mitigate renal toxicity biomarkers and kidney damage by repressing oxidative stress, the NLRP3 inflammasome activation, and apoptosis in CI-AKI by activating autophagy [81]. Recent work has highlighted that inhibition of autophagy can aggravate CI-AKI. Gang Jee Ko and colleagues [82] pointed out that autophagy inhibition by 3-MA aggravates the apoptosis of renal tubule epithelial cells in CI-AKI rats, enhances the oxidative stress caused by contrast agents, and aggravates the kidney damage induced by contrast agents.

### Epigenetic regulation of miRNAs

MicroRNAs (miRNAs) are a class of non-coding, single-stranded RNA molecules with a length of about 22 nucleotides encoded by endogenous genes, which are involved in the regulation of post-transcriptional gene expression [83]. More than 1,000 miRNAs have been identified in various diseases, including CI-AKI [84]. Gutiérrez Escolano et al. [85]performed the earliest study on miRNAs in CI-AKI, and found 17 kinds of differentially expressed miRNAs in kidney tissues through microarray analysis. Liu et al. [86] found that 19 significantly up-regulated and 22 significantly down-regulated miRNAs in the kidneys of CI-AKI rat model.

A series of reports using in vitro cellular experimental and in vivo animal models of CI-AKI have shown MiRNA can regulate downstream gene to influence ICM-induced apoptosis, pyroptosis, and autophagy. As an example, miR-188 is significantly upregulated in both CI-AKI rats and contrast agents-induced HK-2 cells, and the overexpression of miR-188 significantly regulates SRSF7 to promote apoptosis of renal tubular epithelial cells [87]. Niu et al. [88]confirmed that miR-429 up-regulation induced by iodixanol could target PDCD4 to inhibit NF-κB signalling pathway and thus attenuates apoptosis to alleviate CI-AKI. Documents had also unveiled that the expression of miR-30e-5p is up-regulated in CI-AKI, and overexpressed miR-30e-5p represses autophagy and promotes apoptosis by reducing the level of one target gene - Beclin1(autophagy associated protein), thereby contributing to CI-AKI [89]. In miniature pigs, miR-30c up-regulation induced by iohexol could alleviates CI-AKI renal injury through diminishing pyroptosis via inhibiting the activation of the NLRP3 inflammasome [90].

In addition, miRNAs can be readily detected in plasma and serum in a remarkably stable form. The expression profiles of circulating miRNAs carry immense potential for their use as novel, noninvasive biomarkers in diagnosing and monitoring human diseases [91]. As an example, compared with healthy controls, plasma levels of miR-188, miR-30a and miR-3e are significantly elevated in patients with CI-AKI, implying the roles of these miRNAs as promising biomarkers for the diagnosis of CI-AKI [92]. Although the study of miRNAs in CI-AKI is still in its infancy, these datas suggest that miRNAs can be used as potential targets for CI-AKI therapy and biomarkers for early diagnosis.

**Table 2:** Mechanism of the toxic effects of iodine contrast medium and the related ROS

Types	Possible mechanisms	Interpretations	Related pathways or gene expression	References
Apoptosis	Oxidative stress induced by ROS is a potent inducer of apoptosis.	Ca^2+^ overload to ROS overproduction	p38 MAPK pathway, endothelin activation	[62]
		Inhibiting Ca^2+^ overload	CaMII-mPTP pathway	[63]
Pyroptosis	Activate a strong inflammatory response.	Anti-inflammatory and antioxidant.	NLRP3-Caspase-1-GSDMD	[68]
		ICM promote the release of inflammatory factors.	NLRP3-Caspase-1-GSDMD	[69]
		Forming pores in cell membrane via GSDMD dissociation.	Caspase-4/5/11	[71]
Autophagy	Selective removal of damaged mitochondria, and protect cells from damage induced by excessive ROS.	Reducing mtROS and inhibiting the activation of the NLRP3 inflammasome to repair damaged mitochondria.	Pink1/Parkin pathway	[77]
		Rapamycin enhancing mitophagy, alleviating mitochondrial damage and oxidative stress caused by ROS overproduction.	Up-regulating Parkin	[78]
		Melatonin activated autophagy, repressed oxidative stress.	Inhibiting the NLRP3 inflammasome activation	[79]
		3-MA inhibits autophagy	Enhances the oxidative stress	[80]
miRNAs	Regulate gene expression in the post-transcriptional level by degrading target mRNAs or inhibiting translation.	Overexpression of miR-188 promotes apoptosis of renal tubular epithelial cells.	SRSF7	[85]
		miR-429 up-regulation inhibits the activation of the NF-ĸB pathway.	PDCD4	[86]
		Overexpressed miR-30e-5p represses autophagy and promotes apoptosis	Beclin1	[87]
		Upregulated miR-30c diminishes pyroptosis can alleviates CI-AKI renal injury.	NLRP3	[88]

## Risk factors of CI-AKI

The risk of developing CI-AKI depends on factors related to the patient, the type, dose and the route of administration of ICM, and the medication. The details of risk factors of CI-AKI are as follows in Table 3.

### Patient-related risk factors

The correlation between age and CI-AKI is still unclear. The 2019 ACR guidelines [93] stated that patients over the age of 60 need to assess their renal function before receiving ICM. However, the 2018 ESUR [5] did not consider advanced age as a risk factor for CI-AKI. The association between the risk of CI-AKI and advanced age might be due to the fact that patients often experience renal dysfunction or other comorbidities as they age. Therefore, it cannot be confirmed that age is an independent influencing factor for CI-AKI.

Compared with patients with normal kidney function, patients who had CKD before using ICM have a significantly increased risk of developing CI-AKI if they undergo ICM examination. The 2018 ESUR guidelines [5] proposed that eGFR < 45 ml · min^− 1^ · (1.73 m^2^) ^−1^ for arterial injection of ICM or eGFR < 30 ml · min^− 1^ · (1.73 m^2^) ^−1^ for intravenous injection of ICM as independent risk factors for CI-AKI based on the route of administration of ICM.

Diabetes is a common risk factor for CI-AKI. By evaluating the risk of CI-AKI in patients undergoing coronary angiography or coronary intervention, it was found that the incidence of CI-AKI in patients with diabetes was higher [94]. However, diabetes patients have more diseases, so it is not clear whether diabetes is an independent risk factor for CI-AKI.

At present, some studies have found that high serum uric acid levels may be related to the occurrence of CI-AKI. A cohort study involving 1440 patients showed that serum uric acid levels ≥ 8.0 mg/dl were associated with an increased risk of CI-AKI [95].

### ICM-related risk factors: type, dose and the route of administration

Due to the important role of the physicochemical properties of ICM (mainly osmotic concentration and viscosity) in their nephrotoxicity [41]. Multiple clinical trials and meta-analyses have shown no evidence that iso-osmolar ICM are associated with a significantly lower rate of CI-AKI than non-ionic, low-osmolar ICM [96]. However, when ionic, high-osmolar ICM are used, the risk of CI-AKI is increased [97].

The nephrotoxic effects of ICM may be proportional to the dosage used, and the use of higher doses of ICM is associated with an increase in the incidence and mortality of CI-AKI [98]. Repeated ICM administration within a short interval (48–72 h) has been shown to increase the risk of CI-AKI [99].

Although the 2018 ESUR Guidelines [5] proposed that arterial injection of ICM has a higher risk of CI-AKI compared to intravenous injection, no prospective RCTs have confirmed this association. The current clinical trial conclusions evaluating the risk of CI-AKI due to different administration routes are inconsistent.

### Medication-related risk factors

Many frequently prescribed medications, such as nonselective NSAIDs, selective Cox-2 inhibitors, several classes of antimicrobial agents and chemotherapeutic agents have nephrotoxic potential and can induce AKI. The risk of CI-AKI was significantly increased [100], so the 2018 ESUR proposed that the use of nephrotoxic drugs withshould be minimized when clinically possible [8]. 2020 the ACR and National Kidney Foundation (NKF)consensus similarly proposed [6], patients with the combined use of nephrotoxic drugs should detect Scr levels before and after the use of ICM. For patients who have already developed an AKI or an eGFR < 30 ml · min^− 1^ · (1.73 m^2^) ^−1^, the use of non-essential nephrotoxic drugs and drugs that can affect kidney function is not recommended within 48 h before and after the use of ICM.

Metformin is a clinical first-line antidiabetic drug, and its use combined with ICM has a potential risk of lactic acidosis. However, there are differences between the need to stop metformin, how to restart after the instructions and different guidelines [8].

**Table 3:** Risk factors of CI-AKI

Types	Related risk factors	Examples	Guide or conclusions
Patient-related risk factors	Elderly population	> 60 yr	The 2019 ACR
		Did not consider advanced age as a risk factor.	The 2018 ESUR
		eGFR < 45 ml · min^− 1^ · (1.73 m^2^) ^−1^ for arterial injection of ICM.	The 2018 ESUR
		eGFR < 30 ml · min^− 1^ · (1.73 m^2^) ^−1^ for intravenous injection of ICM.	The 2018 ESUR
		Especially with associated renal disease.	Diabetes patients have more diseases, so it is not clear diabetes is an independent risk factor for CI-AKI.
Kidney function (CKD or recent AKI)	High serum uric acid	≥ 8.0 mg/dl	1440 patients showed that high serum uric acid were associated with an increased risk of CI-AKI.
	Type	Ionic, high-osmolar ICM	The risk of CI-AKI is increased.
Diabetes	Dose	High doses of ICM	The use of higher doses of ICM is associated with an increase in the incidence and mortality of CI-AKI.
	The route of administration	Arterial injection of ICM has a higher risk of CI ⁃ AKI compared to intravenous injection.	The 2018 ESUR
Medication-related risk factors	Concurrent nephrotoxic drugs	Including nonselective NSAIDs, selective Cox-2 inhibitors, etc.	The risk of CI-AKI was significantly increased with the combined use of nephrotoxic drugs.
		Detect Scr levels before and after the use of ICM.	The 2018 ESUR
	Metformin	A potential risk of lactic acidosis with ICM. But did not consider metformin as a risk factor.	The 2018 ESUR

## Prevention and treatment of CI-AKI

Although the incidence of CI-AKI is gradually increasing, effective prevention therapy of CI-AKI are very limited at present. Based on the potential pathogenesis of CI-AKI, the commonly used approaches for prevention and treatment of CI-AKI are as follows in Table 4.

8.1 Dilution of concentration, reduction of viscosity, acceleration of excretion, and reduction of retention time of contrast agents.

Hydration therapy is currently recognized as an effective measure to prevent CI-AKI, which is the only proven effective preventive strategies as opposedto other pharmacologic interventions none of which is proven effective. It can reduce the risk of CI-AKI by improving renal blood flow, dilute the ICM in the renal tubules, reducing the activation of the renin angiotensin system, and reducing the secretion of antidiuretic hormone. Therefore, both the 2012 KDIGO AKI Clinical Practice guideline [7] and the 2018 EUSR CI-AKI prevention guidelines [8] recommend CI-AKI prevention because of hydration therapy. According to the 2016 Chinese Guidelines for Percutaneous Coronary Intervention, isotonic saline hydration therapy has been used as an effective approach for CI-AKI prevention in patients with moderate to severe chronic kidney disease [101].

Both intravenous and oral hydration may reduce the risk of CI-AKI. In contrast to intravenous hydration, oral hydration prevents CI-AKI due to difficulty in monitoring or controlling hydration speed, the 2018 EUSR guidelines [8] does not recommend the use of oral hydration as the preferred or sole prevention strategy for CI-AKI and does not limit oral hydration in addition to the preferred intravenous hydration.

Saline (0.9% NaCl) and bicarbonate, sodium solution (1.4% or 154 mmol/L NaHCO_3_) are the most studied intravenous hydrated solutions. But saline and sodium bicarbonate solutions have not uniformly prevented the differences in the effectiveness of CI-AKI. The recent PRESERVE trial studies [102] demonstrated that intravenous sodium bicarbonate versus intravenous sodium chloride had no significant benefit in preventing death, need for dialysis or sustained decline in renal function or CI-AKI in high-risk patients undergoing angiography. Therefore, the intravenous injection of hydration of bicarbonate has similar effectiveness with the hydration of normal saline, but we should consider the high cost of bicarbonate solution and the possibility of alkalosis, so as to select appropriate hydration crystals for patients.

At present, there is no consensus on the optimal hydration regimen (speed, volume, time, etc.). Due to the differences in the risk of CI-AKI and hydration conditions between inpatients and outpatients, individualized adjustments should be made during hydration [8].

### Inhibition of ROS production, oxidative stress, and inflammatory response

As previously discussed, ICM administration could cause an increase of ROS production in vasa recta and tubule cells and consequently induces apoptosis, pyroptosis and autophagy activation. Several compounds with antioxidant properties have been investigated including N-acetylcysteine (NAC), statins, and Vitamin C.

For instance, these purposes can be achieved by the use of the anti-oxidant NAC. NAC was the direct scavenger of free radicals and is thought to have a protective effect on CI-AKI by reducing ROS production, improving blood flow and dilating blood vessels through NO-mediated pathways [103]. Heyman et al. [27]showed that the anti-oxidant NAC could prevent and treat CI-AKI by reducing ROS production and attenuating oxidative stress and inflammatory response.

However, recent RCTs or meta-analyses did not show a preventive effect of NAC against coronary or peripheral angiography [104] or CI-AKI [105] for in patients undergoing enhanced CT. The recent PRESERVE trial studies [102] also showed that in patients at high risk of renal disease undergoing angiography, oral NAC did not reduce from death, requiring dialysis, persistent renal function decline after 90 d, or the risk of developing CI-AKI. The effectiveness of the NAC in preventing CI-AKI remains undefined.

Statins, suppressors of hydroxymethylglutaryl coenzyme A, are recently very popular drugs for CI-AKI prevention. Statins can alleviate kidney injury by reducing the formation of local oxygen free radicals and renal ischemia/hypoxia state, thereby reducing the risk of CI-AKI. Moreover, studies have shown that statin therapy can effectively reduce contrast-induced AKI [106].Despite many positive results, it is difficult to make a generally recommended [107] for statins, as these studied patients were cardiac and used multiple statins and standard hydration regimens, eGFR < 45 ml · min^− 1^ (1.73 m^2^) ^−1^ patients were not included and most patients undergoing coronary angiography or coronary intervention were already on statins for a long time, these confounding factors contributed to uncertainty in results [108]. Therefore, although short-term high-dose statins may be potentially preventive, they is not recommended to prevent CI-AKI in the absence of other indications [8].

SGLT2i are new generation of hypoglycemic drugs used in the treatment of patients with diabetes mellitus type 2 (T2DM). These inhibitors function by specifically blocking the reabsorption of glucose in the renal tubules, leading to increased glucose excretion and lower blood glucose levels [9, 109]. Emerging data suggested that SGLT2i might prevent CI-AKI through mechanisms not directly related to glucose-lowering, such as anti-inflammatory, and anti-oxidative, thus potentially preventing and attenuating some CI-AKI pathological pathways [110, 111]. Huang. et al. [112] investigated that dapagliflozin(one of the SGLT2i)may ameliorate CI-AKI. On the cell and a rat model established by iohexol, it was clear that dapagliflozin significantly reduced hypoxic injury, apoptosis and kidney histopathological damages by attenuating the HIF-1 alfa/human epididymis protein (HE4)/nuclear factor kappa B (NF-kB) signaling pathway.Moreover, a latest research has shown that the potential protective effect of SGLT2i against CI-AKI in patients with diabetes [113]. Despite some positive results, a very limited body of evidence has focused on the potential link between CI-AKI and SGLT2i therapy. Therefore, although SGLT2i may be potentially preventive, there is not recommended to prevent CI-AKI in the absence of other indications such as T2DM.

Due to the antioxidant properties of Vitamin C, its efficacy in the prevention of oxidative stress-associated diseases has been studied extensively. Vitamin C is a safe, well-tolerated, and readily available antioxidant. Some studies have reported that Vitamin C has a preventive effect on CI-AKI [114]. Some meta-analysis also proved that Vitamin C combined with normal saline can significantly reduce the risk of CI-AKI [115]. However, most RCTs, studies or meta-analyses have not demonstrated that Vitamin C reduces the risk of CI-AKI in patients who received coronary CKD angiography [105]. Vitamin C may have a potential and a preventive effect for CI-AKI, but it still needs to be confirmed by clinical studies.

### Improvement of renal perfusion, replenishment of blood volume, and improvement of ischemia/hypoxia state

Adenosine is a vasoconstrictive nucleoside of the kidney, and contrast agents can activate adenosine receptors to cause hemodynamic changes in the kidney, thereby leading to CI-AKI. Theophylline, an adenosine receptor antagonist, is capable of significantly reducing the incidence of CI-AKI and the level of creatinine [116]. However, there are uncertain results regarding the effects of theophylline on CI-AKI [117, 118], nor should theophylline alone be recommended to reduce the risk of CI-AKI.

In order to avoid the occurrence of CI-AKI, effective prevention is very important. A precise knowledge of the dosage, indication, and use time of contrast agents is thus essential for CI-AKI prevention. The diminishment of the dosage and the avoidance of repeated use of contrast agents in the short term are also very crucial for the reduction of CI-AKI. The risk factors for CI-AKI, such as chronic renal insufficiency, diabetes, advanced age, cardiovascular disease, hemodynamic instability, and some drug combinations should always be noted.

**Table 4:** Summary of the commonly used approaches for prevention and treatment of CI-AKI

Approaches	Modes of action	Administration route	Dispute
Hydration therapy	Improving renal blood flow, diluting the ICM, reducing the renin angiotensin system, and reducing the secretion of antidiuretic.	Intravenous, Oral	1.Does not recommend the use of oral hydration as the preferred or sole prevention strategy for CI-AKI. 2.There is controversy over the use of bicarbonate.
NAC	Scavenger of free radicals, vasodilation, precursor for glutathione synthesis, inhibit angiotensin-converting enzyme.	Oral	Recent RCTs or meta-analyses did not show a preventive effect of NAC against CI-AKI.
Statins	Pleiotropic action, improving endothelial function, maintaining nitric oxide production and reducing free radicals formation through NADPH oxidase activity.	Oral	Patients were cardiac and used multiple statins and standard hydration regimens, these confounding factors contributed to uncertainty in results.
SGLT2i	Anti-inflammatory, and anti-oxidative	Oral	There is not recommended to prevent CI-AKI in the absence of other indications such as T2DM.
Vitamin C	Scavenger of oxygen free radicals.	Oral	Most RCTs have not demonstrated that Vitamin C reduces the risk of CI-AKI.
Theophylline	An adenosine receptor antagonis.	Oral	Uncertain results regarding the effects of theophylline on CI-AKI.

## Conclusions

The definition of CI-AKI clearly indicates a causal relationship between the use of ICM and the sharp decline in renal function. The incidence of CI-AKI is lower in the general population, but significantly higher in the high-risk population. Iodine contrast medium can directly induce nephrotoxicity, cause changes in hemodynamic viscosity, and enhance oxidative stress-induced ROS. Apoptosis, pyroptosis, mitophagy, and some epigenetic regulatory factors, such as microRNAs (miRNAs), are associated with the cytotoxic effects of iodine contrast medium and induced ROS. Based on the above mechanism, it may lead to the occurrence and development of CI-AKI. Identifying high-risk comparison subjects and controlling for related risk factors is crucial. Reasonable and effective hydration is a key measure for preventing and treating CI-AKI, and the efficacy of new preventive and therapeutic drugs needs further exploration. The continuous development of medical technology and the deeper understanding of CI-AKI pathogenesis would provide new opportunities to design better interventions for effective prevention and treatment in CI-AKI.

## Data Availability

The data that are available from the corresponding author upon reasonable request.
